# Assessing renal interstitial fibrosis using compartmental, non-compartmental, and model-free diffusion MRI approaches

**DOI:** 10.1186/s13244-024-01736-2

**Published:** 2024-06-20

**Authors:** Wentao Hu, Yongming Dai, Fang Liu, Tianshu Yang, Yao Wang, Yiwei Shen, Wenyan Zhou, Dongmei Wu, Leyi Gu, Minfang Zhang, Yan Zhou

**Affiliations:** 1 Department of Radiology, Renji Hospital, Shanghai Jiao Tong University School of Medicine, Shanghai, China; 2 School of Biomedical Engineering, ShanghaiTech University, Shanghai, China; 3 Department of Nephrology, Renji Hospital, Shanghai Jiao Tong University School of Medicine, Shanghai, China; 4 Shanghai Key Laboratory of Magnetic Resonance, School of Physics and Electronics Science, East China Normal University, Shanghai, China

**Keywords:** Renal interstitial fibrosis, Diffusion relaxation correlated spectrum imaging, Intra-voxel incoherent motion, Diffusion kurtosis imaging, Corticomedullary difference

## Abstract

**Objective:**

To assess renal interstitial fibrosis (IF) using diffusion MRI approaches, and explore whether corticomedullary difference (CMD) of diffusion parameters, combination among MRI parameters, or combination with estimated glomerular filtration rate (eGFR) benefit IF evaluation.

**Methods:**

Forty-two patients with chronic kidney disease were included, undergoing MRI examinations. MRI parameters from apparent diffusion coefficient (ADC), intra-voxel incoherent motion (IVIM), diffusion kurtosis imaging (DKI), and diffusion-relaxation correlated spectrum imaging (DR-CSI) were obtained both for renal cortex and medulla. CMD of these parameters was calculated. Pathological IF scores (1–3) were obtained by biopsy. Patients were divided into mild (IF = 1, *n* = 23) and moderate-severe fibrosis (IF = 2–3, *n* = 19) groups. Group comparisons for MRI parameters were performed. Diagnostic performances were assessed by the receiver operator’s curve analysis for discriminating mild from moderate-severe IF patients.

**Results:**

Significant inter-group differences existed for cortical ADC, IVIM-D, IVIM-f, DKI-MD, DR-CSI *V*_B_, and DR-CSI *V*_C_. Significant inter-group differences existed in ΔADC, ΔMD, Δ*V*_B_, Δ*V*_C_, Δ*Q*_B,_ and Δ*Q*_C_. Among the cortical MRI parameters, *V*_B_ displayed the highest AUC = 0.849, while ADC, *f*, and MD also showed AUC > 0.8. After combining cortical value and CMD, the diagnostic performances of the MRI parameters were slightly improved except for IVIM-D. Combining *V*_B_ with *f* brings the best performance (AUC = 0.903) among MRI bi-variant models. A combination of cortical *V*_B_, ΔADC, and eGFR brought obvious improvement in diagnostic performance (AUC 0.963 vs 0.879, specificity 0.826 vs 0.896, and sensitivity 1.000 vs 0.842) than eGFR alone.

**Conclusion:**

Our study shows promising results for the assessment of renal IF using diffusion MRI approaches.

**Critical relevance statement:**

Our study explores the non-invasive assessment of renal IF, an independent and effective predictor of renal outcomes, by comparing and combining diffusion MRI approaches including compartmental, non-compartmental, and model-free approaches.

**Key Points:**

- Significant difference exists for diffusion parameters between mild and moderate-severe IF.
- Generally, cortical parameters show better performance than corresponding CMD.
- Bi-variant model lifts the diagnostic performance for assessing IF.

**Graphical Abstract:**

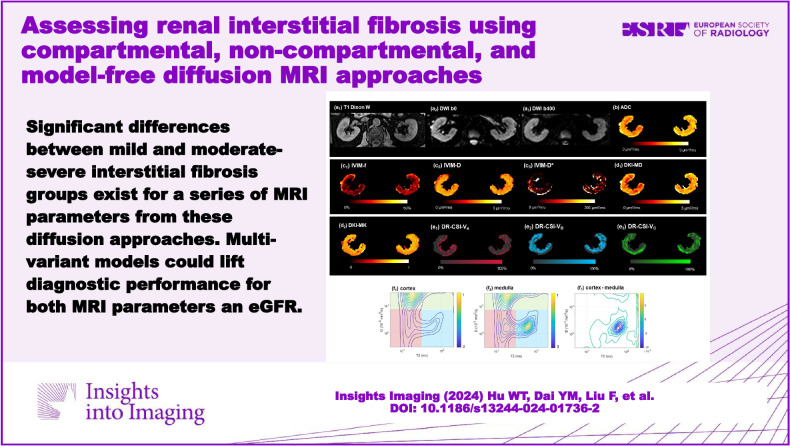

## Introduction

Chronic kidney disease (CKD) is a rising public health concern, defined as the loss of renal function for a long period of time [1]. The global prevalence of CKD is estimated to be 8–16% [2]. A key histological feature of CKD is the presence of interstitial fibrosis (IF) [3], featured by the accumulation of extracellular matrix (ECM) in the renal interstitium and linked to pathologic changes like capillary obliteration and tubular atrophy [2, 4, 5]. Estimated glomerular filtration rate (eGFR) reflects the renal function at a certain time, but is not an independent predictor for renal function impairment. In comparison, IF serves as an independent and effective predictor of renal outcomes [6]. Patients with extensive fibrosis are more likely to progress to end-stage renal failure [7, 8], and may require planning for dialysis or transplantation. Unfortunately, IF usually progresses silently with no explicit manifestations [9]. Biopsy currently remains the only established standard for IF assessment. However, the invasive nature, risk of bleeding, sample bias, and unsuitability for longitude monitoring restrict its clinical value [9]. Non-invasive renal IF assessment methods would certainly benefit CKD diagnosis and treatment monitoring, even though they serve a complementary role.

Among various imaging strategies, diffusion-weighted imaging (DWI) and its derivatives stand out for assessing renal microstructure [10–13]. Fibrotic tissues hinder water mobility in extracellular space, which can be captured by DWI. Past research has already linked the apparent diffusion coefficient (ADC) with IF levels and eGFR [12]. Moreover, fibrosis-related obstructions like increased collagens would deflect the molecular movement further from the so-called Gaussian diffusion [5]. These non-Gaussian properties could be captured not by mono-exponential ADC, but in principle by advanced diffusion models, including compartmental and non-compartmental ones [13]. Intra-voxel incoherent motion (IVIM) is a representative of the former, considering both pseudo- and true-diffusion compartments, frequently utilized in studies of renal fibrosis or dysfunction [14–16]. Decreased IVIM parameters, including *D* and *f*, were reported in IF induced by IgA nephritis or unilateral ureteral obstruction [15, 16]. Conversely, diffusion kurtosis imaging (DKI) represents the non-compartmental model, describing features at high *b* values by an empirical formula, and was increasingly applied in recent CKD studies [17, 18]. A negative correlation between renal parenchymal mean diffusivity and a positive correlation between mean kurtosis to histopathological fibrosis score was reported [17].

Despite the promising findings of diffusion models, they heavily rely on artificial constraints, including predetermined components or specific mathematical formulas. Also, these approaches ignore the influence of other properties like relaxation time, limiting their ability to resolve intra-voxel contributions [19]. Recent advancements have introduced model-free methods like multi-dimensional correlation magnetic resonance imaging (MRI) [20]. A notable example, diffusion-relaxation correlated spectrum imaging (DR-CSI) has preliminarily been applied to kidney evaluation [21, 22]. Fresh insights were provided by incorporating both the T2 dimension and the peak-based spectrum quantification.

Although the aforementioned methods are all based on diffusion MRI, they are established with different hypotheses, and focus on distinct renal features. Therefore, comparison and combination of them for assessing the pathologic changes could be worthy of attempts. Moreover, their assistance to renal functional biomarkers such as eGFR would also be helpful. Besides, although reports frequently suggest that IF progression correlates with cortical MRI measurement [23, 24], some studies also indicate that corticomedullary difference (CMD), like ∆*T*1 and ∆ADC, are effective [3, 10, 25]. Given the assumption that pathologic changes in cortical and medullary microstructure are distinct, it deserves curiosity whether the CMD of these approaches would bring additional interest.

The objective of this study is to (1) assess renal IF using diffusion-based MRI approaches including ADC, IVIM, DKI, and DR-CSI; (2) explore whether CMD of diffusion parameters benefits IF evaluation; and (3) explore whether a combination among these methods or with eGFR could benefit IF evaluation.

## Materials and methods

This cross-sectional study is part of an ongoing prospective research plan on characterizing the longitude alterations of pathologic changes in CKD patients using MRI, approved by the Internal Review Board of our hospital. Written informed consent was obtained.

### Study subjects

From March 2022 to May 2023, fifty consecutive patients suspected of high risk of CKD (with related clinical symptoms lasting for over 3 months and abnormal blood test results) while meeting the following standard were enrolled and underwent renal MRI: (1) aged 18–80 years; (2) no medical history of renal surgery or other significant intervention; and (3) appropriate for the time-extended MRI, evaluated by the on-site medical staff. In the further analysis, eight patients were excluded due to: (1) unavailable of essential clinical and pathological data (*n* = 2); (2) finally diagnosed as (or with comorbid) acute renal injury (*n* = 1); (3) finally diagnosed as end-stage renal disease (stage V) (*n* = 2); (4) incomplete MRI acquisition (*n* = 1); and (5) poor MR image quality (*n* = 2).

### Clinical and pathological evaluation

The eGFR was determined using serum creatine based on the CKD epidemiology collaboration formula [26]. Renal IF was assessed on a biopsy specimen by the Department of Pathology of our hospital. Percutaneous biopsy was performed within 3 days after the MRI examination, and the specimens were stained using Masson’s trichrome method. IF scores were quantified as 1, 2, and 3 based on the percentage of fibrosis < 25%, 25–50%, and > 50%, respectively, according to the Oxford Classification of IgA nephropathy 2016 [27]. For further analysis, patients with an IF score of 1 were classified into the “mild IF” group, while patients with IF 2 or 3 were classified into the “moderate-severe IF” group. This grouping criteria is based on two reasons: the worse prognosis for both moderate and severe IF patients compared to mild ones [28], and the relatively small sample size of IF = 3 patients (*n* = 6). Pathologic diagnosis is given in Table 1.

**Table 1:** Clinical characteristics of the participants in this study

Characteristics	Total, *n* = 42
Age, year	
≤ 30	4
30–40	9
40–49	8
50–59	9
60–69	8
≥ 70	4
Gender	
Male	23
Female	19
CKD stage	
I	13
II	14
III	13
IV	2
IF score	
1 (Mild)	23
2 (Moderate)	13
3 (Severe)	6
Primary clinicopathologic diagnosis	
IgA nephropathy	16
Membranous nephropathy	10
Lupus nephritis	3
Podocyte injury	2
Diabetic nephropathy	2
Thrombotic micro-angiopathy	2
Renal arteriosclerosis	3
Glomerulosclerosis	1
Glomerulonephritis	1
Hepatitis B associated nephritis	1
Light chain deposition	1

*CKD* chronic kidney disease, *IF* interstitial fibrosis

### MRI acquisition

All participants underwent examination on a 3.0-T MRI scanner (Magnetom Prisma, Siemens Healthineers) using an 18-channel phase-array body coil and embedded spine coil. MRI protocols included: axial T1-weighted (T1w) Dixon, axial fat-suppressed T2-weighted (T2w), coronal T2w, axial multi-b DWI, and axial DR-CSI. The multi-b DWI scan was realized by a spin-echo single-shot echo-planar-imaging (SE-SS-EPI) sequence with three directions and 12 *b* values: 0_1_ s/mm^2^, 10_1_ s/mm^2^, 30_1_ s/mm^2^, 50_1_ s/mm^2^, 70_1_ s/mm^2^, 100_1_ s/mm^2^, 200_1_ s/mm^2^, 400_1_ s/mm^2^, 800_2_ s/mm^2^, 1000_3_ s/mm^2^, 1500_4_ s/mm^2^, 2000_5_ s/mm^2^, 2500_6_ s/mm^2^, respectively (the subscript denotes for the average). Other parameters are: echo time (TE) 54 ms, repetition time (TR) 2100 ms, field of view (FOV) 380 × 283 mm^2^, acquisition matrix 135 × 100, interpolation factor 2, 10 slices, slice thickness/gap 3.0/3.0 mm. The average was taken for the three orthogonal directions, and the diffusion anisotropy was ignored. The DR-CSI scan was realized by a SE-SS-EPI sequence with 36 acquisitions: six TEs (51–200 ms) combined with six *b* values (0–1500 s/mm^2^). Other parameters of the DR-CSI protocol, including TR, FOV, matrix, and slice thickness/gap, were kept the same to the multi-b DWI sequence. The detailed MR protocols are listed in Table 2.

**Table 2:** The detailed MR protocols used in this study

	T2w BLADE with FS	T2w HASTE	T1w VIBE Dixon	Multi-b DWI	Multi-TE-multi-b DWI
Plane	Transverse	Coronal	Transverse	Transverse	Transverse
TR (ms)	3300–8000	400	3.97	2100	2100
TE (ms)	86	96	1.29	54	51, 80, 110, 140, 180, 200
FOV (mm^2^)	380 × 380	400 × 400	380 × 308	380 × 283	380 × 283
Acquisition matrix	384 × 384	320 × 256	320 × 182	135 × 100	135 × 100
Slice thickness/gap (mm)	4.0/1.2	4.0/0.4	3.0/0.0	3.0/3.0	3.0/3.0
Num of slice	35	30	72	10	6
Bandwidth (Hz/pixel)	710	710	1040	2330	2330
*b* values (s/mm^2^)	/	/	/	0, 10, 30, 50, 70, 100, 200, 400, 800, 1000, 1500, 2000, 2500	0, 150, 400, 800, 1200, 1500
Method of acquisition	Belt trigger	Breath-hold	Breath-hold	Free-breathing	Free-breathing
Scan time	2–4 min	12 s	2 × 12 s	3 min 54 s	9 min 18 s

*TR* repetition time, *TE* echo time, *FOV* field of view, *T1w* T1-weighted, *T2w* T2-weighted, *DWI* diffusion-weighted imaging, *FS* fat saturation, *TSE* turbo spin echo, *BLADE* proprietary name for periodically rotated overlapping parallel lines with enhanced reconstruction (PROPELLER), *HASTE* half-Fourier acquired single-shot turbo spin-echo, *VIBE* volumetric interpolated breath-hold examination

### Image post-processing

Region of interest (ROI) is decided for cortex and medulla separately, including all slices displaying kidney. Two radiologists (with 7 years and 4 years of experience in abdominal MRI, respectively) manually delineated the ROI on DWI b0 with the assistance of T1w images (Fig. S1) by using the segment editor tool embedded in 3D-Slicer (https://www.slicer.org/). Attempts were made to avoid focal areas. An average cortical area of 105.3 cm^2^ and medulla area of 37.9 cm^2^ were obtained for the 12-b DWI sequence, while an average cortical area of 71.8 cm^2^ and medulla area of 26.6 cm^2^ were obtained for DR-CSI. ADC mapping was fitted using b0 and b800 images from the 12-b DWI sequence.

### IVIM fitting

IVIM and DKI models were fitted voxel-by-voxel using homemade scripts on MATLAB, derived from the 12-b DWI sequence. IVIM model is defined as follows:

$$\frac{S\left(b\right)}{{S}_{0}}\,=\,f\cdot \exp \left(-b\cdot D\right)\,+\,(1\,-\,f)\cdot \exp \left(-b\cdot {D}^{* }\right)$$

Where ${S}_{0}$ is the original signal intensity, *D* and D* are the diffusion coefficients of normal- and pseudo-diffusion components, and *f* is the pseudo-diffusion fraction. In renal MRI, the selection of the *b* value may influence the results of diffusion models. Therefore, attempts were made to separate the non-Gaussian effect caused by perfusion and parenchyma. For IVIM analysis, *b* values utmost to 800 s/mm^2^ were selected to avoid the kurtosis effect at high *b* values. A two-step fitting strategy was applied setting the threshold of 200 s/mm^2^ [29].

### DKI fitting

DKI model adopted in this study is a direction-averaged model, which is recommended in body MRI [30]:

$$S\left(b\right)/{S}_{0}\,=\,\exp \left(-b\cdot {MD}\,+\,\frac{1}{6}{MK}\cdot {b}^{2}\cdot {{MD}}^{2}\right)$$

Where MD is the mean diffusivity, and MK is the mean kurtosis. To minimize the potential perfusion effect and concentrate on soft-tissue-induced complexity, *b* values from 200 s/mm^2^ to 2500 s/mm^2^ were selected, while low *b* values were excluded. Similar practices could be found in previous research assuming the large perfusion in the kidney [31].

### DR-CSI

The principle of DR-CSI has been illustrated clearly in the literature [20, 32]. In practice, a discrete form of DR-CSI is adopted:

$${S}_{i}\left(b,{T}_{E}\right)\,=\,{\sum}_{j,k}{f}_{i}\left({D}_{j},{T2}_{k}\right)* {e}^{-b\cdot {D}_{j}}* {e}^{-{TE}/{T2}_{k}}$$

Where ${f}_{i}$ is spectral intensity representing the distribution on a D-T2 mesh, while each mesh point represents a “component” with specific diffusivity and relaxometry. Specifically, the mesh consists of 30 rows of D (log-spaced, 0.3–30 μm^2^/ms) and 30 columns of T2 (log-spaced, 5–200 ms). The DR-CSI post-processing workflow is similar to the previous paper [21]. Particularly, three compartments A (short T2), B (long T2), and C (high diffusivity) were defined by boundaries manually chosen (T2 boundary within 30–50 ms, diffusivity boundary 6–9 μm^2^/ms) at a visible peak gap. Thus, DR-CSI volume fractions *V*_X_ for compartment X were obtained by a summation over “components”. Compartments A and B were considered to relate to intra- and extra-cellular water, while C was considered to link with microvascular and body liquid flow [21].

### CMD

**Fig. 1:**
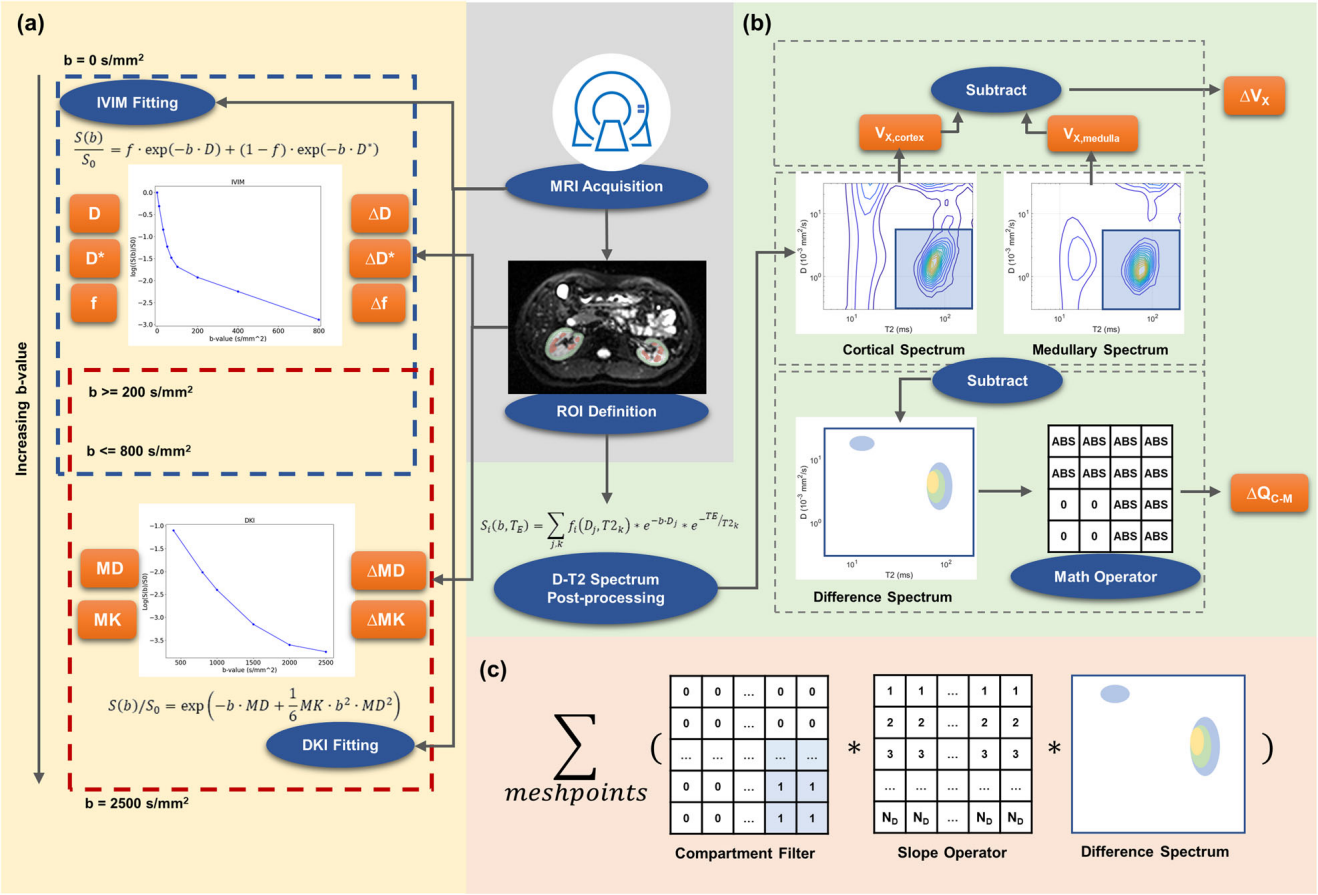
Workflow of the main post-processing procedures. **a** IVIM and DKI fitting using DWI of different ranges of *b* value. CMD was obtained for each individual by subtraction of the mean value in cortex and medulla. **b** DR-CSI post-processing includes two types of CMD. The traditional one simply calculates the volume fraction for the cortex and medulla separately, and performs a subtraction. The new one is obtained by applying a mathematical operator on the cortex-minus-medulla spectrum. **c** The mathematical operator used in this study. Intensity on the D-T2 spectrum was multiplied by a compartmental filter and order operator, and then summed up. IVIM, intra-voxel incoherent motion; DKI, diffusion kurtosis imaging; DR-CSI, diffusion-relaxation correlated spectrum imaging; ROI, region of interest; MD, mean diffusivity; MK, mean kurtosis

For common MRI parameters, a CMD of a patient was defined as the average value of cortex ROI minus the average value of medullary ROI. The notation “Δ” is used as an abbreviation of CMD before a parameter name (e.g. ΔADC). In addition to traditional DR-CSI CMD (Δ*V*_X_), a novel “spectral CMD” (Δ*Q*_X_) was defined (Fig. 1): a difference-spectrum was obtained by subtracting the normalized medullary D-T2 spectrum from the cortical one, and then filtered by a spectral operator. Δ*Q*_B_ and Δ*Q*_C_ were calculated due to the effectiveness of compartments B and C in evaluating IF [21]. A detailed description was given in Supplementary Material.

### Statistics

All statistical analysis was conducted using SPSS (v23.0; IBM Corp.). The significance criteria were *p* < 0.05 throughout this study. Intraclass correlation coefficient (ICC) was applied to assess the inter-observer agreement of the MRI parameters. ICC < 0.6, 0.6–0.8, and > 0.8 were defined as poor, fair, and good, separately. If the agreement reached fair or good, the value measured by the more experienced radiologist was adopted. Spearman’s test was used to evaluate the correlation between (1) MRI parameters and (2) each MRI parameter to eGFR. Group comparison was conducted using student’s *t*-test (age, eGFR), Mann–Whitney *U-*test (MRI parameters), or Chi-square test (gender).

Multivariable linear regression models were established to identify moderate-severe IF from mild IF, including the combination of (1) MRI cortical parameters and corresponding CMD, (2) several MRI parameters, and (3) eGFR and MRI parameters. Several principles were obeyed to choose the appropriate MRI parameter, with details in Supplementary Material (“construction of multivariant models” section). Accordingly, cortical *V*_B_, ADC *f*, and ∆ADC were considered in the multi-variant models. The diagnostic performance of parameters (or models) was evaluated using receiver operator’s curve (ROC) analysis, with the area under the curve (AUC), sensitivity, and specificity (optimized by Youden’s Index) calculated. AUC values < 0.6, 0.6–0.7, 0.7–0.8, 0.8–0.9, and > 0.9 were interpreted as poor, acceptable, moderate, good, and excellent diagnostic performance.

## Results

### Clinical characteristics

**Fig. 2:**
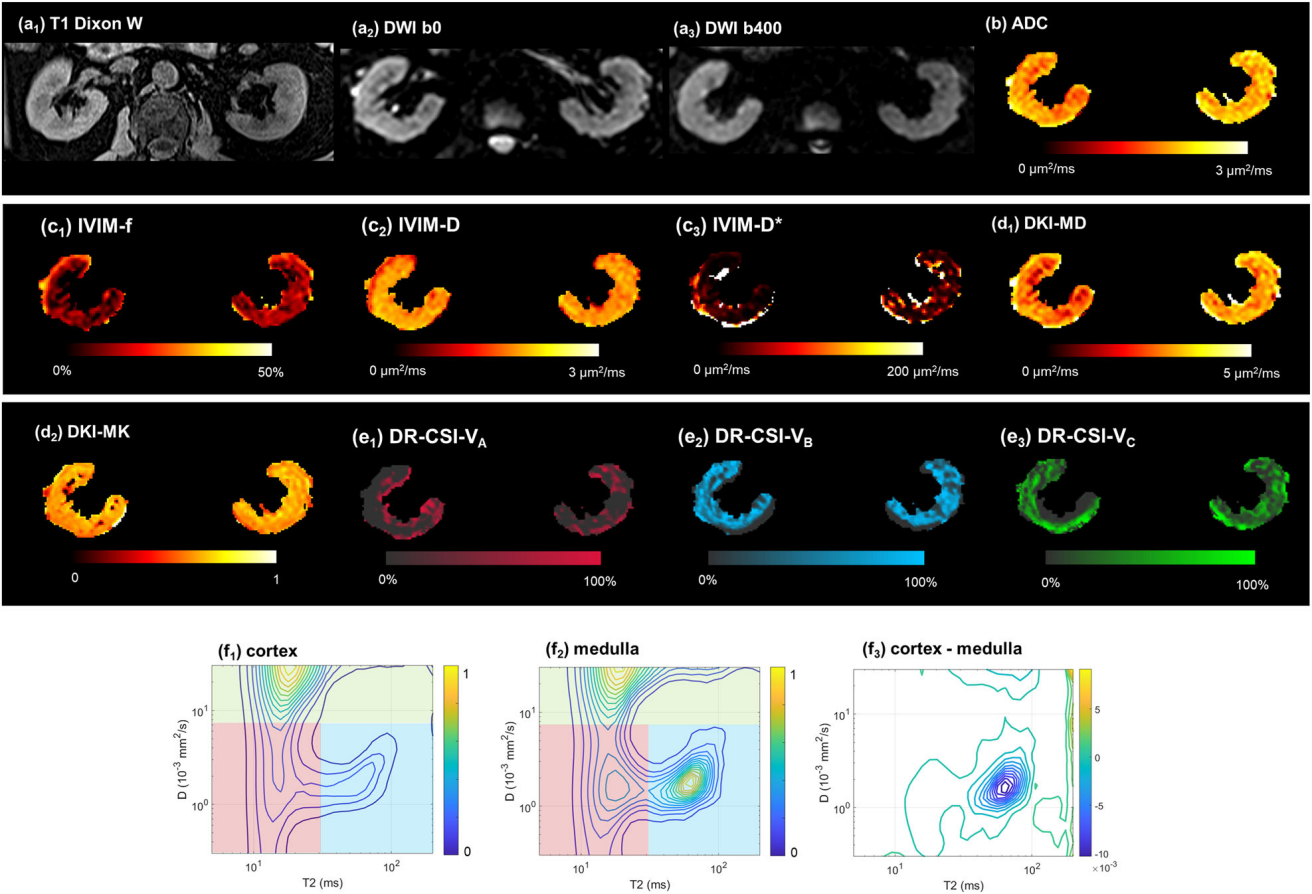
A 65-year-old male with membranous nephropathy, pathologically confirmed as mild IF. **a**_**1**_–**a**_**3**_ Cortex displayed hyperintensity compared to medulla on T1w DIXON water and DWI b0, but less distinguishable on DWI b400. **b**–**e** Parameter mappings by (**b**) ADC, (**c**_**1**_–**c**_**3**_) IVIM, (**d**_**1**_, **d**_**2**_) DKI, and (**e**_**1**_–**e**_**3**_) DR-CSI were given. **f**_**1**_–**f**_**3**_ D-T2 spectrum by DR-CSI of the cortex, medulla, and their difference. CKD, chronic kidney disease; IVIM, intra-voxel incoherent motion; DKI, diffusion kurtosis imaging; DR-CSI, diffusion-relaxation correlated spectrum imaging; MD, mean diffusivity; MK, mean kurtosis

**Fig. 3:**
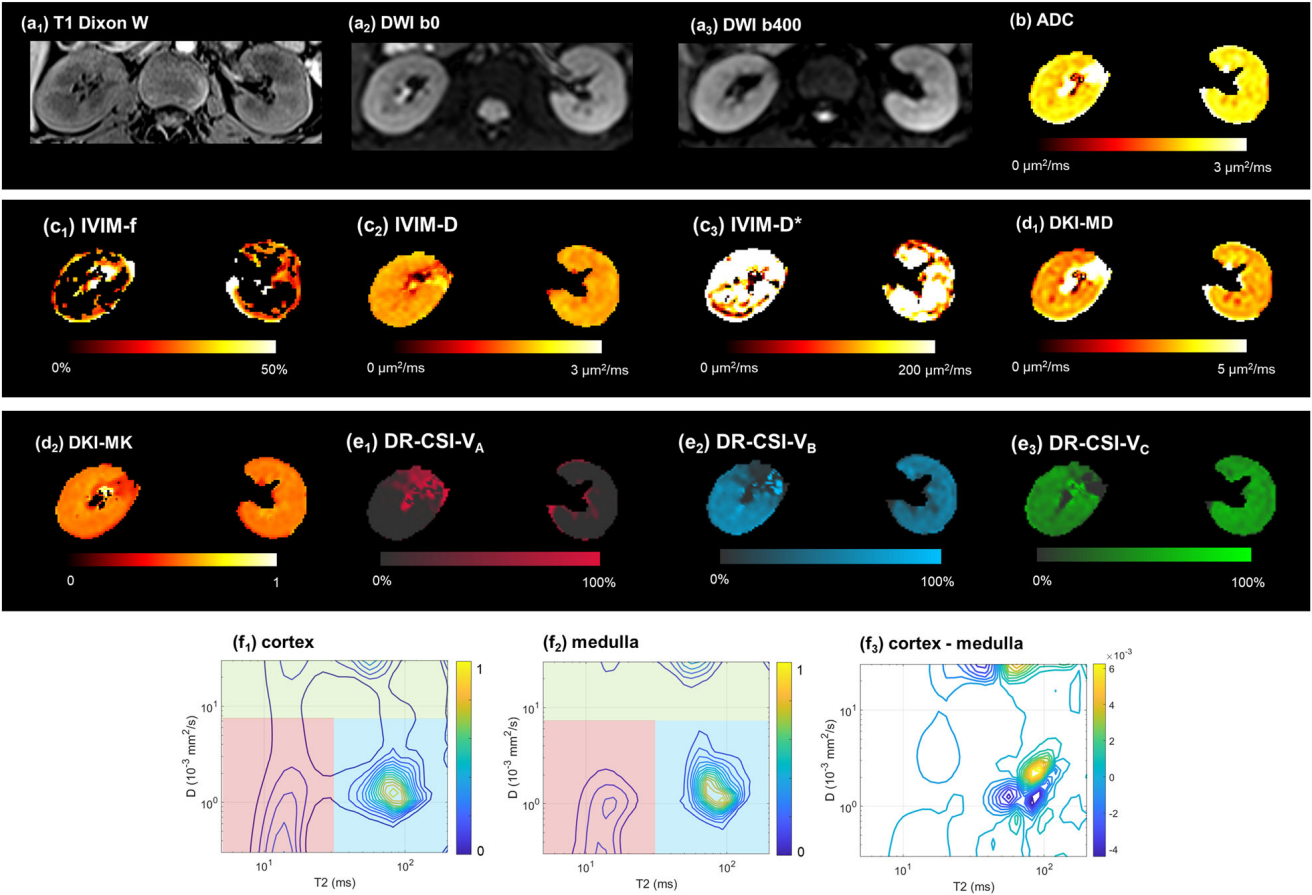
A 24-year-old female with IgA nephritis, pathologically confirmed as severe IF. **a**_**1**_–**a**_**3**_ Cortex displayed hyperintensity compared to medulla on T1w DIXON water, but was less distinguishable on both DWI b0 and b400. **b**–**e** Parameter mappings by **b** ADC, **c**_**1**_–**c**_**3**_ IVIM, **d**_**1**_, **d**_**2**_ DKI, and **e**_**1**_–**e**_**3**_ DR-CSI were given. **f**_**1**_–**f**_**3**_ D-T2 spectrum by DR-CSI of cortex, medulla, and their difference. CKD, chronic kidney disease; IVIM, intra-voxel incoherent motion; DKI, diffusion kurtosis imaging; DR-CSI, diffusion-relaxation correlated spectrum imaging; MD, mean diffusivity; MK, mean kurtosis

Finally, data from 42 CKD patients (23 male, 19 female, age 24–74) were adopted into analysis, including 23 mild, 13 moderate, and 6 severe IF patients. Two typical cases are given in Figs. 2 and 3. Detailed clinical information is given in Table 1. No significant differences were found in age (*p* = 0.726) or sex (*p* = 0.801) between the mild and moderate-severe IF groups. The moderate-severe IF group had a significantly lower eGFR (51.1 ± 24.7 vs 90.3 ± 19.7, *p* < 0.001).

### Group comparison

Inter-operator agreements for all parameters are fair to good (Table S1). The results of the group comparison for MRI parameters are listed in Table 3. Within the region of the cortex, significant differences could be found for ADC, *D*, *f*, MD, *V*_B,_ and *V*_C_, among the two groups. Specifically, ADC (1.97 ± 0.23 vs 2.19 ± 0.12 μm^2^/ms, *p* = 0.001), D (1.54 ± 0.16 vs 1.65 ± 0.10 μm^2^/ms, *p* = 0.019), *f* (17.3 ± 4.9% vs 22.6 ± 4.6%, *p* = 0.001), MD (2.74 ± 0.48 vs 3.17 ± 0.29 μm^2^/ms, *p* = 0.001), and *V*_C_ (24.9 ± 5.3% vs 31.0 ± 6.5%, *p* = 0.003) presented lower values, while *V*_B_ (54.5 ± 8.4% vs 42.9 ± 8.5%, *p* = 0.001) displayed higher value in the moderate-severe IF compared to the mild IF. Within the region of the medulla, significantly higher D* (67.4 ± 17.1 vs 53.1 ± 19.5 μm^2^/ms, *p* = 0.018) and lower *f* (12.1 ± 3.3% vs 16.1 ± 4.2%, *p* = 0.002) were found in the moderate-severe group compared to the mild group. For most parameters, the moderate-severe group tended to have less CMD compared to the mild group. A significant difference could be found in ΔADC (0.19 ± 0.10 vs 0.33 ± 0.14 μm^2^/ms, *p* < 0.001), ΔMD (0.48 ± 0.23 vs 0.77 ± 0.31 μm^2^/ms, *p* = 0.002), Δ*V*_B_ (0.5 ± 8.6% vs −7.3 ± 7.9%, *p* = 0.007), Δ*V*_C_ (2.9 ± 4.6% vs 6.6 ± 5.8%, *p* = 0.037), Δ*Q*_B_ (0.01 ± 1.17 vs −1.15 ± 1.16, *p* = 0.007) and Δ*Q*_C_ (0.39 ± 0.89 vs 1.28 ± 1.24, *p* = 0.016).

**Table 3:** Group comparison of cortical, medullary values, and CMDs of diffusion MRI-derived parameters

	IF = 1	IF = 2-3	*p* value
Mono-exponential			
Cortical ADC (μm^2^/ms)	2.19 ± 0.12	1.97 ± 0.23	0.001^**^
Medullary ADC (μm^2^/ms)	1.85 ± 0.12	1.78 ± 0.16	0.123
ΔADC (μm^2^/ms)	0.33 ± 0.14	0.19 ± 0.10	< 0.001^***^
IVIM			
Cortical *D* (μm^2^/ms)	1.65 ± 0.10	1.54 ± 0.16	0.019^*^
Cortical D* (μm^2^/ms)	73.0 ± 20.3	77.9 ± 26.6	0.579
Cortical *f* (%)	22.6 ± 4.6	17.3 ± 4.9	0.001^**^
Medullary *D* (μm^2^/ms)	1.64 ± 0.09	1.57 ± 0.12	0.090
Medullary D* (μm^2^/ms)	53.1 ± 19.5	67.4 ± 17.1	0.018^*^
Medullary *f* (%)	16.1 ± 4.2	12.1 ± 3.3	0.002^**^
Δ*D* (μm^2^/ms)	0.01 ± 0.08	−0.03 ± 0.10	0.137
ΔD* (μm^2^/ms)	20.0 ± 19.0	10.4 ± 20.9	0.126
Δ*f* (%)	6.5 ± 5.1	5.2 ± 3.7	0.356
DKI			
Cortical MD (μm^2^/ms)	3.17 ± 0.29	2.74 ± 0.48	0.001^**^
Cortical MK	0.492 ± 0.029	0.502 ± 0.040	0.376
Medullary MD (μm^2^/ms)	2.39 ± 0.26	2.26 ± 0.32	0.181
Medullary MK	0.483 ± 0.059	0.487 ± 0.057	0.850
ΔMD (μm^2^/ms)	0.77 ± 0.31	0.48 ± 0.23	0.002^**^
ΔMK	0.008 ± 0.049	0.014 ± 0.046	0.668
DR-CSI			
Cortical *V*_A_ (%)	26.0 ± 7.3	20.9 ± 8.3	0.067
Cortical *V*_B_ (%)	42.9 ± 8.5	54.5 ± 8.4	0.001^**^
Cortical *V*_C_ (%)	31.0 ± 6.5	24.9 ± 5.3	0.003^**^
Medullary *V*_A_ (%)	25.7 ± 4.9	23.6 ± 8.7	0.283
Medullary *V*_B_ (%)	50.1 ± 8.3	54.9 ± 9.4	0.240
Medullary *V*_C_ (%)	24.3 ± 5.8	22.0 ± 5.5	0.346
Δ*V*_A_ (%)	−0.2 ± 6.4	−2.7 ± 7.6	0.153
Δ*V*_B_ (%)	−7.3 ± 7.9	0.5 ± 8.6	0.007^**^
Δ*V*_C_ (%)	6.6 ± 5.8	2.9 ± 4.6	0.037^*^
Δ*Q*_B_	−1.15 ± 1.16	0.01 ± 1.17	0.007^**^
Δ*Q*_C_	1.28 ± 1.24	0.39 ± 0.89	0.016^*^

Δ Represents the CMD, which was defined as cortical value minus medullary value

Significant level: * *p* < 0.05, ** *p* < 0.01, *** *p* < 0.001

*IF* interstitial fibrosis, *ADC* apparent diffusion coefficient, *IVIM* intra-voxel incoherent motion, *DKI* diffusion kurtosis imaging, *MD* mean diffusivity, *MK* mean kurtosis, *DR-CSI* diffusion relaxation correlated spectrum imaging

### Correlations between MRI parameters and eGFR

**Fig. 4:**
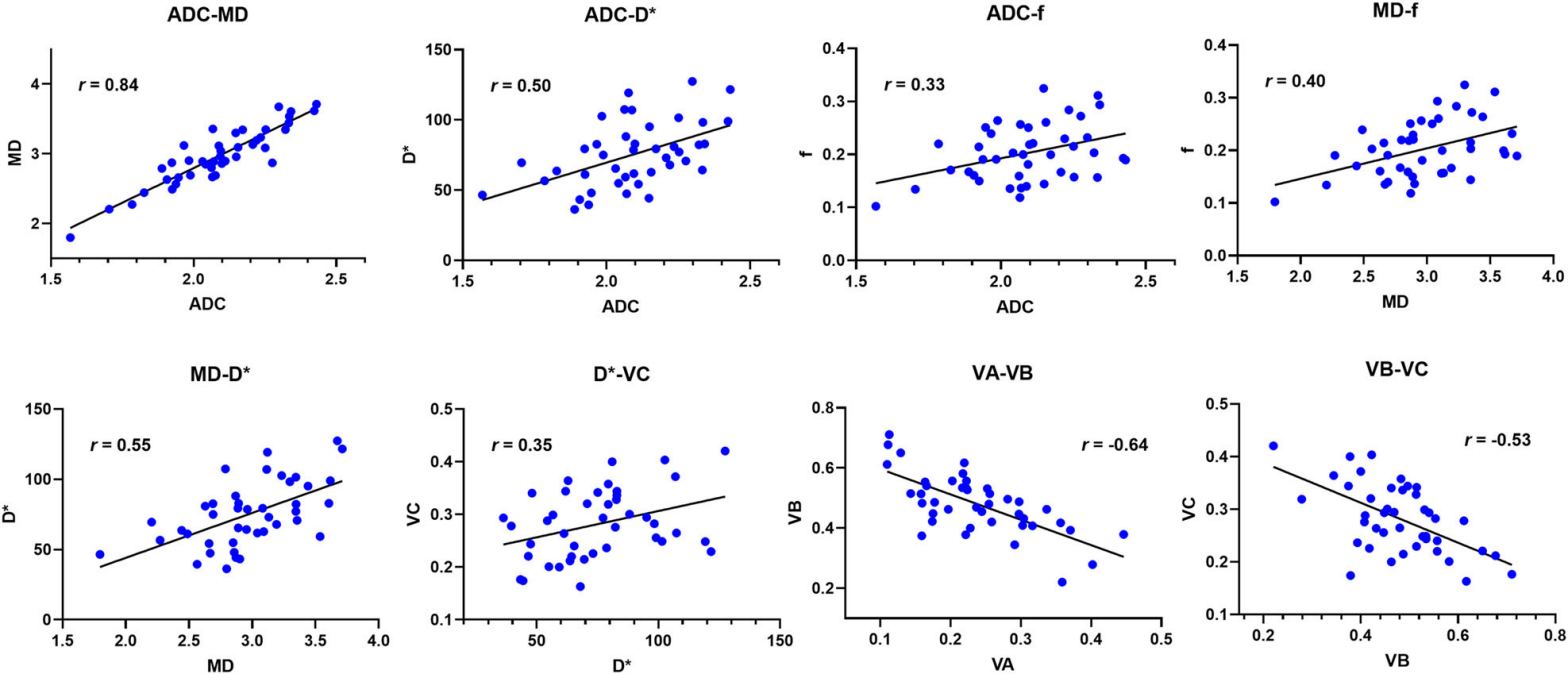
The heat-map plot illustrates Spearman’s correlation coefficient (*r*) among cortical MRI parameters. ADC, apparent diffusion coefficient; MD, mean diffusivity; MK, mean kurtosis

The correlations among cortical MRI parameters are presented in Figs. 4 and S2. A strong correlation exists between ADC with MD (*r* = 0.84, *p* < 0.001). A moderate correlation exists between MD with D* (*r* = 0.55), ADC with D* (*r* = 0.50), V_A_ with *V*_B_ (*r* = −0.64), and *V*_B_ with *V*_C_ (*r* = −0.53) (all *p* < 0.001). A significant but weak correlation exists between f with ADC (*r* = 0.33), *f* with MD (*r* = 0.40), and VC with D* (*r* = 0.35) (all *p* < 0.05). Among the correlation within medullary parameters (Figs. S3 and S4), similar relationships could be found, despite the significant correlations between ADC with D (*r* = 0.41) and between D* with MK (*r* = 0.43) (both *p* < 0.01).

The correlations for MRI parameters to eGFR are presented in Fig. S5. Positive correlations exist in cortical ADC (*r* = 0.45), *D* (*r* = 0.62), *f* (*r* = 0.33), MD (*r* = 0.36), *V*_C_ (*r* = 0.49), medullary *D* (*r* = 0.50), *f* (*r* = 0.32), and ΔADC (*r* = 0.39), ΔMD (*r* = 0.36), and Δ*V*_C_ (*r* = 0.39). A negative correlation exists in cortical *V*_B_ (*r* = −0.45).

### Assessing IF

**Fig. 5:**
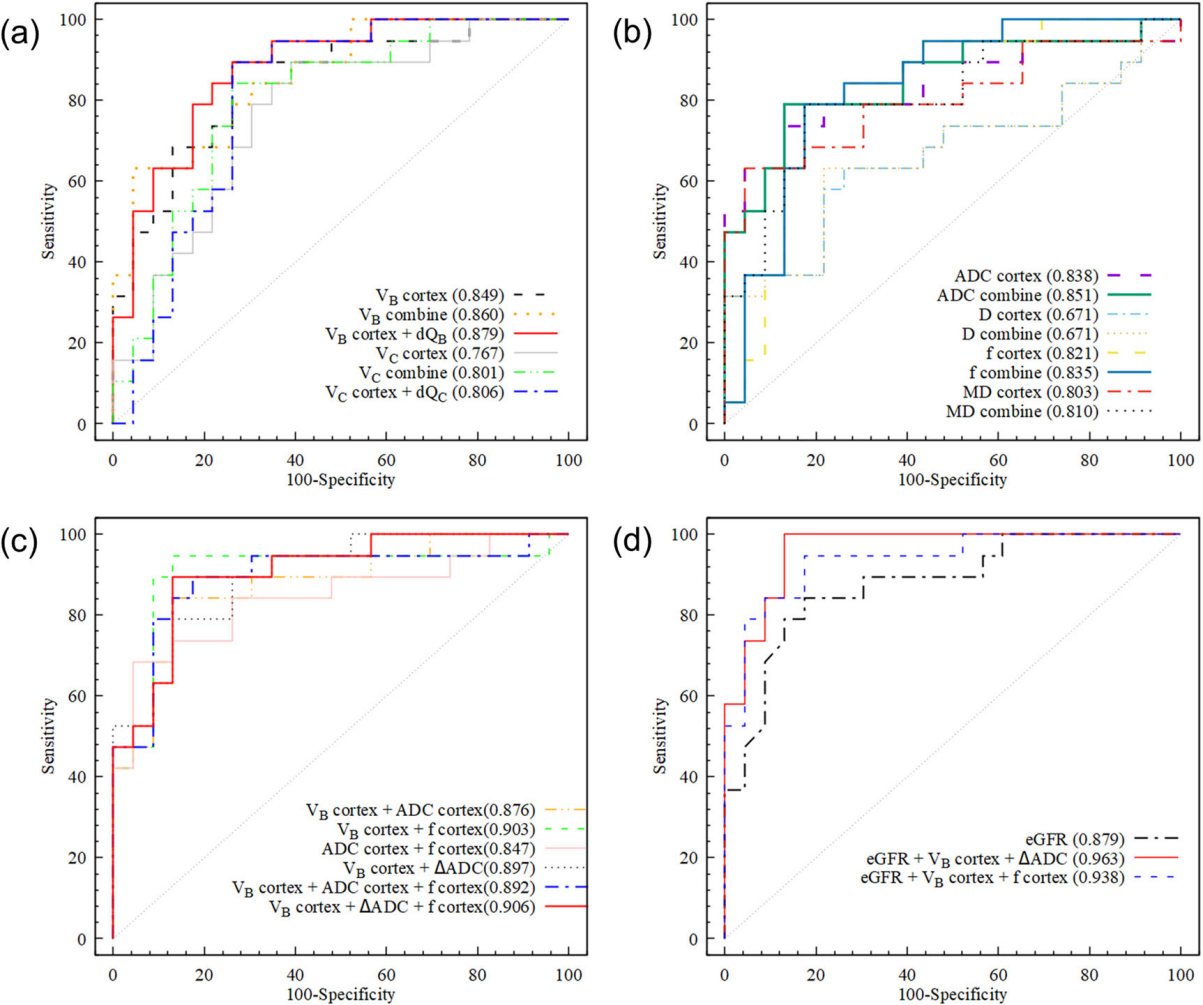
ROC curves discriminating moderate-severe fibrosis with (**a**) cortical DR-CSI parameters and combination to their CMD; **b** DWI model parameters and combination to their CMD; **c** multivariant MRI model; **d** eGFR and combination to MRI parameters. ROC, receiver operator’s curve; CMD, corticomedullary difference; DWI, diffusion-weighted imaging; DR-CSI, diffusion-relaxation correlated spectrum imaging; eGFR, estimated glomerular filtration rate; ADC, apparent diffusion coefficient; MD, mean diffusivity

The results of ROC analysis are presented in Tables 4 and 5 and Fig. 5. Cortical parameters showed a higher AUC than CMD for all parameters. Among the cortical MRI parameters, DR-CSI *V*_B_ showed a numerically highest AUC (0.849), while ADC, *f*, and MD also showed good performance (AUC 0.803–0.838). Among the CMD of parameters, only ΔADC showed good performance (AUC = 0.828).

**Table 4:** Diagnostic performance of MRI parameters for the discrimination of moderate-severe IF from mild IF

	Cortex				CMD				Combination^a^			
	AUC	Cut-off	Specificity	Sensitivity	AUC	Cut-off	Specificity	Sensitivity	AUC	Cut-off	Specificity	Sensitivity
ADC (μm^2^/ms)	0.838	2.065	0.869	0.737	0.828	0.263	0.739	0.894	0.851	2.147, 0.142	0.870	0.790
IVIM *D* (μm^2^/ms)	0.671	1.579	0.739	0.632	0.609	−0.036	0.739	0.526	0.671	1.580, 0.036	0.783	0.632
IVIM *f* (%)	0.821	19.10	0.826	0.789	0.618	9.28	0.391	0.947	0.835	18.93, 2.91	0.783	0.842
DKI MD (μm^2^/ms)	0.803	2.798	0.956	0.631	0.792	0.508	0.783	0.790	0.810	2.955, 0.610	0.826	0.790
DR-CSI *V*_B_ (%)	0.849	46.87	0.739	0.894	0.764	−3.05	0.696	0.790	0.860	54.05, −1.13	0.957	0.632
DR-CSI ∆*Q*_B_	/	/	/	/	0.769	−0.075	0.826	0.632	0.879	48.03, −0.86	0.826	0.842
DR-CSI *V*_C_ (%)	0.767	29.44	0.609	0.894	0.736	4.99	0.652	0.842	0.801	26.50 7.58	0.739	0.842
DR-CSI ∆*Q*_C_	/	/	/	/	0.719	1.69	0.739	0.631	0.806	22.94 1.69	0.739	0.894

^a^ Combined linear regression model using cortical value and CMD of MRI parameter

Δ Represents the CMD, which was defined as cortical value minus medullary value

*IF* interstitial fibrosis, *CMD* corticomedullary difference, *ADC* apparent diffusion coefficient, *IVIM* intra-voxel incoherent motion, *DKI* diffusion kurtosis imaging, *MD* mean diffusivity, *MK* mean kurtosis, *DR-CSI* diffusion relaxation correlated spectrum imaging

**Table 5:** Diagnostic performance of multivariant models using MRI, eGFR, and multivariant models combined MRI and eGFR, for the discriminating moderate-severe IF from mild IF

	AUC	Specificity	Sensitivity
*V*_B_ + ADC	0.876	0.869	0.842
*V*_B_ + *f*	0.903	0.869	0.947
ADC + *f*	0.847	0.956	0.684
*V*_B_ + ΔADC	0.897	0.870	0.789
*V*_B_ + ADC + *f*	0.892	0.826	0.895
*V*_B_ + ΔADC + *f*	0.906	0.870	0.895
eGFR	0.879	0.826	0.842
eGFR + *V*_B_ + ΔADC	0.963	0.896	1.000
eGFR + *V*_B_ + *f*	0.938	0.826	0.947

MRI parameters in this table are cortical values if not stated

Δ Represents the CMD, which was defined as cortical value minus medullary value

*IF* interstitial fibrosis, *ADC* apparent diffusion coefficient, *eGFR* estimated glomerular filtration rate

After combining cortical value with CMD, AUC and specificity for most of the involved parameters were slightly improved (1–5%), except for *D*. Combining cortical *V*_B_ with ΔQ_B_ (AUC = 0.879) showed slightly better lift than with Δ*V*_B_ (AUC = 0.860). The combination of cortical *V*_C_ with Δ*Q*_C_ reached a larger sensitivity (0.894) than with Δ*V*_C_ (0.842), while the AUC is similar (0.806 vs 0.801).

Combining *V*_B_ with *f* brings the best performance (AUC = 0.903) among MRI bi-variant models, followed by *V*_B_ with ΔADC (AUC = 0.897), both bringing *a* > 5% AUC raise. MRI tri-variant models did not show a large improvement (0.892–0.906). Discriminating moderate-severe IF using eGFR alone turned out a good performance (AUC = 0.879). A combination of cortical *V*_B_, ΔADC, and eGFR could reach the excellent performance of AUC 0.963, as well as a rise of specificity (0.826 vs 0.896) and sensitivity (0.842 vs 1.000) compared to eGFR alone. A combination of cortical *V*_B_, *f*, and eGFR also displayed improved AUC (0.879 vs 0.938) and sensitivity (0.842 vs 0.947) compared to eGFR alone.

## Discussions

Our study compared, as well as combined several diffusion MRI approaches including conventional (ADC), compartmental (IVIM), non-compartmental (DKI), and model-free (DR-CSI) ones for differentiating CKD patients with moderate or severe IF from those with mild fibrosis. Each method revealed promising imaging biomarkers, demonstrating significant differences between groups. Among them, *V*_B_, ADC, ΔADC, *f*, and MD showed good diagnostic capabilities, which were further enhanced using bi-variant models. Moreover, we found that integrating diffusion MRI metrics offered a diagnostic improvement over using eGFR alone.

Pseudo-diffusion compartments in IVIM are believed to link with perfusion, which is an appealing topic in renal research [14, 15, 33]. For severe CKD patients, tubular atrophy often co-exists with IF, leading to an anticipated reduction in perfusion. This is corroborated by our findings, where *f* was one-fourth lower in the more severe IF group within both the cortex and medulla. Although cortical *D* also presented a difference, its diagnostic performance was unexpectedly lower than *f*, contrary to our initial assumption and previous report in immunoglobulin A nephropathy [16]. This discrepancy might be explained by the larger volume of tubular atrophy than interstitium, and is further supported by a stronger correlation for *f* than *D* to histopathological fibrosis score in literature [34]. A known challenge with IVIM is the instability of D* fitting, evidenced by the substantial standard deviation for D* in this study. Also, D* demonstrates the lowest inter-observer agreement, consistent with existing literature [31]. Given that common ADC measurement incorporates a low *b* value (0 s/mm^2^ or 50 s/mm^2^). its correlation to IVIM-f and D* is expected. Among IVIM parameters, our study highlights the clinical relevance of *f*, followed by *D*, while D* is not recommended.

While the adoption of DKI in renal studies is increasing, the interpretation of “complexity” remains ambiguous. Contrary to prior research, we did not find a significant change in MK toward severe fibrosis [35]. Additionally, the comparative kurtosis between cortex and medulla holds higher, and remains a topic of debate, given contradictions in either fibrotic [35, 36] or healthy kidney [31, 37, 38]. Our study reports a slight but positive CMD for both groups of patients. Fortunately, MD is less controversial, as our study demonstrates inter-group differences and good diagnostic performance aligns with the previous findings [35, 36]. However, its additional value to ADC is limited by their similarity in outcomes, as evidenced by the strong Spearman correlation. This is attributed to their overlapping role in measuring mean diffusivity. The clinical utility of DKI in assessing renal IF may need more investigation.

Advanced compartmental models, such as a three-component IVIM model, have been introduced to capture renal microstructure [39]. However, accurately determining these compartments is mathematically challenged due to the difficulties of inverting multi-exponential signal decay [40]. Encouragingly, this ill-posedness is alleviated by an expanded dimension [19, 20], and DR-CSI has showcased its potential in renal microstructure imaging [21]. In this study, cortical *V*_B_ outperformed other parameters, which implies that hindered extra-cellular water may be tightly associated with IF. The newly defined spectral CMD showed promising results, providing the inspiration that model-free approaches may maximize the advantage by leveraging both peak shift and change of fractions. Elevated Δ*V*_B_ and ΔQ_B_ in moderate-severe IF might relate to the increased ECM accumulation. Decreased Δ*V*_C_ and Δ*Q*_C_ hint at a diminished contribution and reduced diffusivity within cortical microvascular. DR-CSI stands out for its less correlation with parameters from other models, likely attributed to the inclusion of T2. This enhanced the diagnostic capability when combining *V*_B_ with *f* or ADC. An exception is *V*_C_ correlated with D*, both representing perfusion-related characters. Nevertheless, inherent negative correlations exist within the DR-CSI fractions since they must add up to 1, underscoring the complexity of interpreting these measurements.

The results of multi-variant models are encouraging, reflecting the diverse biophysical focus of these approaches. Diffusion MRI fulfilled the expectation as a robust adjunct to eGFR, since eGFR could only reflect one, although important, aspect of renal function. Our study reported greater numbers of cortical parameters showing inter-group differences, aligning with the previous opinion that loss of T1w CMD in fibrotic kidneys was raised by cortical changes [25]. Yet, a subtraction by medulla is still meaningful, considering the individual variation of kidney ADC [10, 41]. A trend of CMD towards zero at severe fibrosis was observed, consistent with prior findings on ADC [3, 10]. As indicated by our findings, the CMD of the advanced diffusion approaches is not a superior indicator on its own but promising as a supplementary input. Combining with kidney segmentation techniques, this may bring modest, cost-efficient, enhancement to current CKD assessments.

This study has several limitations. First, sampling bias is a challenge, since patients with severe CKD are more likely to be judged as unsuitable for biopsy and therefore not included. This might also explain the relatively smaller sample of severe fibrosis cases (*n* = 6). Second, the sample size for the three levels of IF is restricted. This also leads to the merging of the moderate and severe IF into one group, which is another limitation in the study design. Although moderate and severe IF patients show worse prognoses compared to mild ones, they have different clinical implications. Third, the pathological assessment of IF utilized a three-point score instead of providing a quantitative measure of fibrotic tissue proportion. A previous study reached a quantitative assessment of fibrosis to a precision of 10% [3], enabling a correlation analysis with MRI parameters. At last, a minor point that deserves notice is that the DR-CSI sequence in this study covers only six transverse slices. Fibrosis resulting from CKD tends to be diffusely distributed, and functional MRI without whole coverage of the kidney is acceptable. Yet as this technique is in an early stage of application, future efforts to explore larger coverage would be valuable.

## Conclusion

In conclusion, our study shows promising results for the assessment of renal IF using diffusion MRI approaches. For most MRI parameters included, significant differences between different IF groups were found in both cortical value and CMD. Several parameters displayed good diagnostic performance discriminating patients with moderate-severe IF from mild ones. Bi-variant MRI model could lift the diagnostic performance. Combining diffusion parameters could bring improvement compared to eGFR alone. These findings could offer insights into non-invasive evaluation strategies for renal pathology.

### Supplementary information

ELECTRONIC SUPPLEMENTARY MATERIAL

## Data Availability

The datasets generated or analyzed during the study are mainly human MR images, and are not publicly available due to the ethics policies of the hospital, but are available from the corresponding author on reasonable request.

## Abbreviations

ADC: Apparent diffusion coefficient
AUC: Area under the curve
CKD: Chronic kidney disease
CMD: Corticomedullary difference
DKI: Diffusion kurtosis imaging
DR-CSI: Diffusion-relaxation correlated spectrum imaging
DWI: Diffusion-weighted imaging
eGFR: Estimated glomerular filtration rate
FOV: Field of view
ICC: Intraclass correlation coefficient
IF: Interstitial fibrosis
IVIM: Intra-voxel incoherent motion
MRI: Magnetic resonance imaging
ROC: Receiver operator’s curve
T1w: T1-weighted
T2w: T2-weighted
TE: Echo time
TR: Repetition time
