# Supplementary material for: Assessing renal interstitial fibrosis using compartmental, non-compartmental, and model-free diffusion MRI approaches
Source: Insights Imaging. 2024 Jun 20;15:156. doi: 10.1186/s13244-024-01736-2 (PMC11189852; doi:10.1186/s13244-024-01736-2)
Supplement: Supplementary file 1 — ELECTRONIC SUPPLEMENTARY MATERIAL [file 13244_2024_1736_MOESM1_ESM.pdf]

Assessing Renal Interstitial Fibrosis Using Compartmental, Non-Compartmental, and Model-Free Diffusion MRI Approaches

ELECTRONIC SUPPLEMENTARY MATERIAL

Novel DR-CSI Corticomedullary Difference

In this study, an operator considering both change of volume fraction and movement of peak position and was defined. The difference-spectrum was multiplied by a compartment filter (1 for component within compartment X; 0 for others) and a diffusivity slope operator (order j of D_j), and then summed up to the new CMD quantity (labeled as ΔQ_X).

Specifically, the consistency of expected peak movement direction with the variation trend of volume fraction should be considered, realized by the choice of diffusivity slope operator, to properly combine these factors. ΔQ_A was not considered in this study due to the lack of significant difference between the two groups. For compartment B, a raise of V_B is expected to be accompanied by a peak movement toward lower diffusivity. Therefore, the slope operator could be defined that the highest *D*-row on the spectrum takes value of 1 while the lowest *D*-row takes N_D. On the other hand, a raise of V_C is expected to be accompanied by a peak movement toward higher diffusivity. Therefore, the slope operator could be defined that the lowest *D*-row on the spectrum takes value of 1 while the highest *D*-row takes N_D.

Slope operator for T2 was not applied according to the following reasons. First, relaxation-time-based metrices such as apparent T2 value were not included in this study. Second, while there is ample literature supporting DWI's efficacy in evaluating renal interstitial fibrosis, there is limited evidence establishing a direct link with T2 [1-3]. Finally, the expected peak movement direction was not straightforward to confirm for compartment C.

Construction of Multivariant Models

Principle 1: Considering the sample size, upmost three variables were included in a model.

Principle 2: MRI parameters with significant inter-group difference and an AUC>0.7 were taken into consideration. Therefore, cortical MK, D, D* and V_A were excluded.

Principle 3: Since cortical values outperforms CMD, and slight lift of performance was achieved by their combination, we allowed only the best-performed CMD parameter, ΔADC, into multi-variant models.

Principle 4: Avoid to include both parameters if they are with intra-parameter correlation $r>0.5$. Considering the strong correlation between ADC and MD, and the better mono-variant diagnostic performance for ADC, we exclude MD from multivariant models. Similarly, V_C is excluded for its correlation with V_B.

Finally, cortical V_B, ADC and f and ΔADC were considered in the multi-variant models.

Supplementary Tables

Table S1 Inter-operator agreements for MRI parameters

	**Cortex**	**Medulla**
**ADC**	0.924	0.935
**D**	0.866	0.914
**D***	0.781	0.725
**F**	0.903	0.860
**MD**	0.917	0.905
**MK**	0.884	0.892
**VA**	0.852	0.866
**VB**	0.906	0.887
**VC**	0.842	0.848

Supplementary Figures

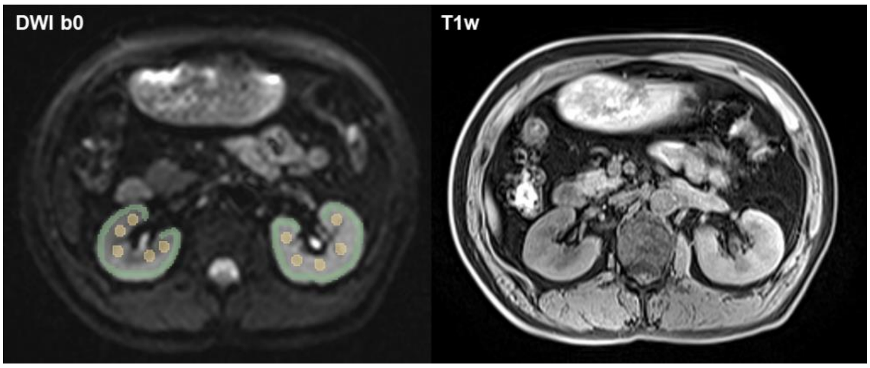

Figure S1. An illustration of region of interest placement. Painting brush with 3 mm width was used to draw cortex region, attempting to exclude boundary voxels to avoid possible partial-volume. Circles of 8 mm diameter were put on medulla with the help of T1w images.

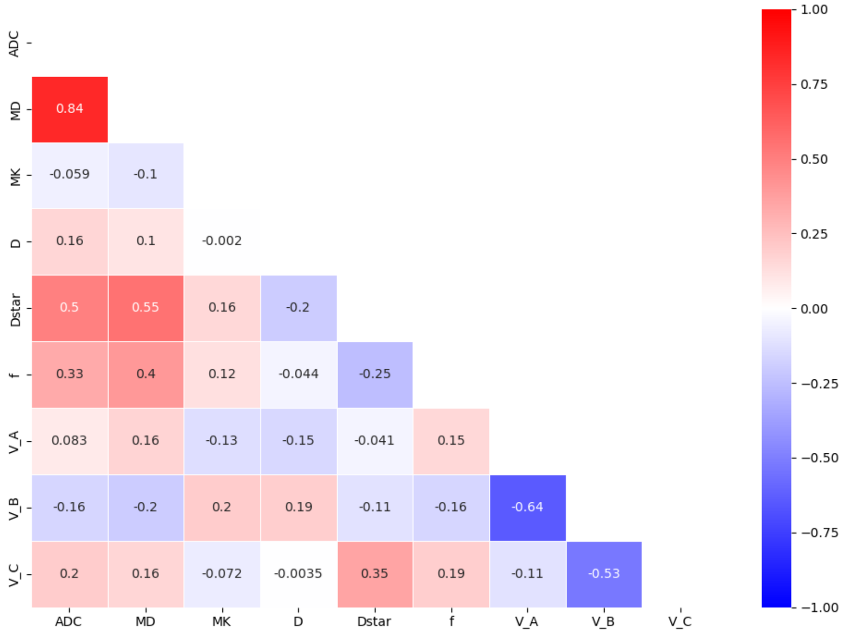

Figure S2. The heat-map plot illustrates the Spearman's correlation coefficient (*r*) among cortical MRI parameters.

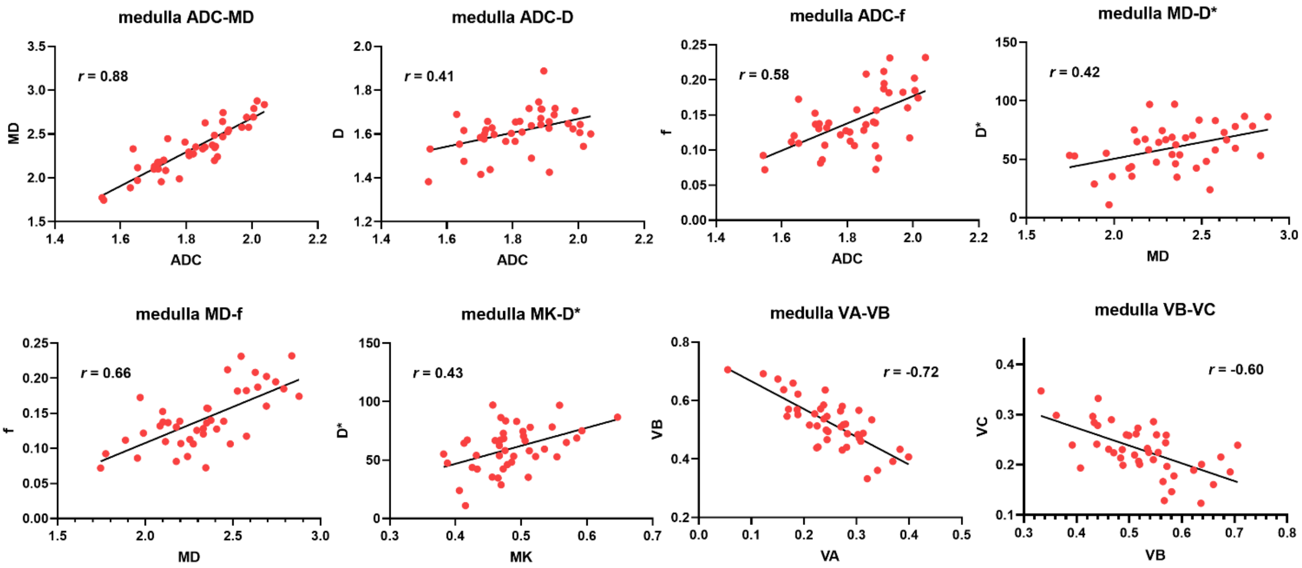

Figure S3. Scatterplots illustrate the significant intra-MRI correlation among medulla parameters. The Spearman's correlation coefficient (*r*) was labelled.

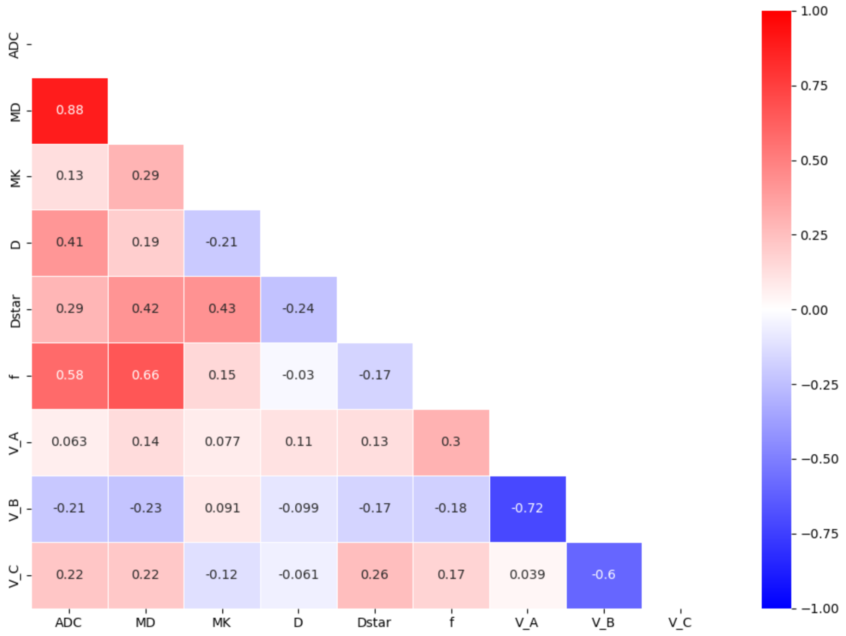

Figure S4. The heat-map plot illustrates the Spearman's correlation coefficient (*r*) among medullary MRI parameters.

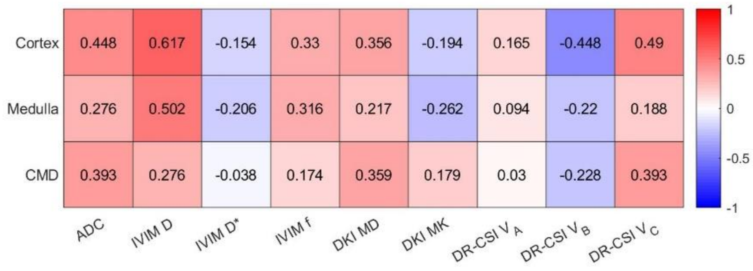

Figure S5. The heat-map plot illustrates the Spearman's correlation coefficient (*r*) for MRI parameters to eGFR.

Reference

1. Jiang K, Ferguson CM, Lerman LO. Noninvasive assessment of renal fibrosis by magnetic resonance imaging and ultrasound techniques. *Translational Research* 2019;209:105-120
2. Bane O, Hectors SJ, Gordic S, Kennedy P, Wagner M, Weiss A, et al. Multiparametric magnetic resonance imaging shows promising results to assess renal transplant dysfunction with fibrosis. *Kidney International* 2020;97:414-420
3. Hueper K, Hensen B, Gutberlet M, Chen RJ, Hartung D, Barrmeyer A, et al. Kidney Transplantation Multiparametric Functional Magnetic Resonance Imaging for Assessment of Renal Allograft Pathophysiology in Mice. *Investigative Radiology* 2016;51:58-65
